# The interactive effects of Indigenous identity and lateral violence on youth adjustment in Aboriginal and Torres Strait Islander children

**DOI:** 10.1080/00049530.2024.2341699

**Published:** 2024-05-09

**Authors:** Taylor-Jai Mcalister, Kris Rogers, Robert Brockman, Gawaian Bodkin-Andrews, John McAloon

**Affiliations:** a Discipline of Clinical Psychology, Graduate School of Health, Faculty of Health, University of Technology Sydney, Ultimo, Australia; b Department of Critical Indigenous Studies, Department of Philosophy, Macquarie University Sydney, Sydney, Australia; c Director of Indigenous Research, Office of the Deputy Vice-Chancellor Indigenous Leadership, Western Sydney University, Sydney, Australia; d UTS: Family Child Behavior Clinic, Discipline of Clinical Psychology, Graduate School of Health, Faculty of Health, University of Technology Sydney, Sydney, Australia

**Keywords:** Aboriginal, Indigenous, First Nations, lateral violence, social and emotional wellbeing, ethnic-racial identity

## Abstract

**Objectives:**

Lateral violence is the potential for members of a group to engage in practices that are harmful to other members of their own group. Evidence indicates that lateral violence can affect Aboriginal children’s social and emotional wellbeing (SEWB); however, little is known about the potential for ethnic-racial identity (ERI) to protect against harmful effects of lateral violence.

**Methods:**

We investigated whether ERI affirmation moderated the relationship between exposure to lateral violence and Aboriginal children’s SEWB. Children (*n* = 360) from the K-Cohort of the Longitudinal Study of Indigenous Children were included in this analysis. Children’s ERI was dichotomised into high versus low affirmation, and General Linear Models were used to examine the effects of lateral violence on SEWB and the potentially moderating effect of ERI on that relationship.

**Results:**

Increased exposure to lateral violence was associated with increased difficulties in all Strengths and Difficulties Questionnaire domains. ERI was also shown to moderate the relationship between exposure to lateral violence and peer difficulties, with children in the high ERI affirmation group showing greater vulnerability than those in the low group.

**Conclusions:**

The findings of this study are discussed in relation to their potential to inform policy and clinical practice.

## Key Points

**What is already known about this topic**:

1. Lateral violence primarily occurs within groups who experience oppression.
2. Questions about a person’s Indigenous status may involve a critique of their authenticity and, therefore, representation of them as more or less Indigenous.
3. Current literature indicates that Ethnic racial identity (ERI) may have positive and negative implications for an individual’s social and emotional wellbeing (SEWB) along several distinct dimensions.

**What this topic adds**:

1. In this study, lateral violence had a significant and negative effect on individual SEWB.
2. Increased exposure to lateral violence was associated with an increase in peer problems, particularly for Indigenous Adolescents who were highly affirming of their ERI.
3. The salience of ERI affirmation may be particularly important for Indigenous adolescents as they seek to establish a clear sense of their own, often unique, identity.

## Introduction

Researchers have been increasingly interested in understanding lateral violence and its impact on Indigenous Australians (Bennett, 2015; Carlson, 2016; Clark et al., 2016). Whilst lateral violence has been portrayed as Aboriginal people receiving questions about the authenticity of their cultural identity from other Aboriginal people, Paakantji scholar Tia Whyman (Whyman et al., 2020, p. 1) reported:

Lateral violence in an Indigenous … context is proposed to occur when colonised peoples internalise their oppressor’s values and behaviours, creating a negative perception of themselves and their culture.

Further, Whyman et al. (2020) noted that lateral violence was expressed physically (e.g., threats and violence), and non-physically through gossip, bullying, shaming, isolation, back-stabbing, denial of identity and adopting non-Indigenous values (see also the seminal Indigenous-led works of Bennett, 2014; Carlson, 2016; Clark et al., 2016; Coffin, 2011; Doyle et al., 2017; Paradies, 2017). From this research, lateral violence can also be regarded as violence turned towards oneself or as a form of internalised colonialism (Dudgeon, 2000). Lateral violence primarily occurs within groups who experience oppression and is therefore inextricably linked to processes of colonisation and assimilation (Dudgeon, 2000; Freire, 1970). Thus, lateral violence may result in questions regarding a person’s Aboriginality, their Indigenous authenticity and categorisations of Indigenous peoples as being *more* or *less* Indigenous (Tatz, 1979).

Work in the area of lateral violence has been developed under a number of frameworks and across a diverse range of populations. For instance, identity denial and self-concept clarity have been explored with respect to their implications for SEWB in bisexual populations (Garr-Schultz & Gardner, 2021), with respect to multiracial identity (Gaither, 2015; Pauker et al., 2018), and based on psychophysiological responses to identity denial in the context of culture and race (Albuja et al., 2019). Thus, lateral violence may be based on personal or physical characteristics. Research has found that Aboriginal people commit lateral violence with members of their own culture or community, particularly in relationship to an individual’s skin-colour or appearance (Bennett, 2014; Boladeras, 2002; Clark et al., 2016; Gorringe et al., 2011).

Literature has also indicated the negative impact of lateral violence on SEWB (Boladeras, 2002; Clark et al., 2016). In the context of Aboriginal and Torres Strait Islander health, the term SEWB accounts for health and wellbeing as it applies to individuals, communities, country and culture, and results from numerous interconnected influences (Dudgeon et al., 2017; Gee et al., 2014; Macedo, Santiago, et al., 2019; Macedo, Smithers, et al., 2019). These include those of self, family, community and the broader social context a person exists in. They include relationship to country, culture, community and kin; the influences of spirituality and ancestors; and physical, emotional and mental health. SEWB is further influenced by social and institutional behaviour, government policy and the enduring effects of colonisation (Dudgeon, 2017; Gee et al., 2014; Macedo, Smithers, et al., 2019).

### Lateral violence

Ballardong/Nyungar scholar Boladeras’s (2002) qualitative research found support for the notion that questions regarding light-skinned Aboriginal people’s authenticity may negatively influence their self-concept and SEWB, leading to ambivalence and self-doubt about their right to identify as Aboriginal. Nyangumarta scholar, Julie Coffin’s (2011) qualitative interviews of Aboriginal children from the Yamaji (Midwest) region of Western Australia reported that bullying from other Aboriginal children was more common and more hurtful than bullying from non-Indigenous children. Researchers have argued that the experience of lateral violence is particularly damaging as it involves a threat to one’s identity and represents a form of cultural rejection from their in-group (C. S. Kickett-Tucker, 2015; Langton, 2008). Further, Wadjuk Noongar scholar C. Kickett-Tucker and Coffin (2010) found pre-adolescents and adolescents may be more vulnerable to the exposure to, and impact of, lateral violence from Aboriginal peers, especially as adolescence is a formative period for exploring and demonstrating one’s Aboriginal identity.

Some researchers have argued that Social Identity Theory (SIT; Tajfel & Turner, 1986) may assist in understanding processes of identity and lateral violence within Indigenous communities (Atkin & Mcalister, 2019; Bennett, 2015; Mcalister & Wheeler, 2018). SIT posits that individuals seek verification as a group member, even for minority groups that may be considered negatively by wider society (North & Swann, 2009). SIT may provide an approach to understanding the impact of experiences of lateral violence on Aboriginal people’s SEWB, through self-categorisation. Self-Categorisation Theory (SCT; Turner, 1991) was developed to further account for the cognitive processes involved in the development of identity, and posits that people define themselves by the categories they belong to. Self-categorisations serve to accentuate perceived similarities between an individual and their in-group, as well as the perceived differences between an individual and the out-group (Turner et al., 1987). Research suggests that skin colour and phenotypical features associated with a minority group are important cues for social categorisation (E. R. Smith & Zarate, 1992; Stepanova & Strube, 2012). Thus, according to SIT and SCT, Aboriginal people who do not possess attributes associated with the social category of *Aboriginal*– for example, skin-colour and phenotypical features – may face challenges because of social categorisation. In this sense, an individual may self-identify with their in-group, however, may be perceived as a member of the out-group.

It is, of course, important to acknowledge that lateral violence may occur in response to a range of characteristics that subgroups within a marginalised population may or may not possess or may or may not have access to. Evidence demonstrates that both context (Bombay et al., 2014; Moodie et al., 2022) and population-based characteristics (Jaber et al., 2023; Webster & Clark, 2024) can be important in determining the experience of lateral violence. Despite an emerging research base in this area, quantitative research involving children and young people and their experience of lateral violence is sparse, and the impact of lateral violence on the SEWB of Aboriginal children and young people remains unclear. Work in the area is largely retrospective, limited to small samples, often qualitative and characterised by demonstrable characteristics (Bombay et al., 2014). What is clear is that a significant proportion of research published in the area with First Nations populations in Australia reports on perceptions of skin colour and the “percentage” of Indigeneity a person may possess (Jaber et al., 2023, p. 1773), despite lateral violence taking many forms including gossip, shaming, verbal and physical assault and bullying (Clark et al., 2017). Importantly, Australia’s specific socio-political context – in particular, policies that aimed to biologically assimilate Aboriginal people (Wolfe, 2006) – may indicate that perceptions of skin-colour and phenotypical features are salient when discussing lateral violence.

### Ethnic-racial identity

Extending existing qualitative research that offers evidence for the negative impact of lateral violence on Aboriginal peoples’ health and SEWB (Bennett, 2015; Boladeras, 2002; Clark et al., 2016), the current study focused on ethnic-racial identity (ERI) as a potentially moderating factor in the relationship between lateral violence and SEWB. Within the literature on minority groups, researchers have debated the use of the terms “racial identity” and “ethnic identity” (Cokley, 2007; Schwartz et al., 2014). This has resulted in the use of the term “ethnic-racial identity” (Schwartz et al., 2014; Umana-Taylor et al., 2014), a term that combines experiences that are not uniquely associated with race or ethnicity, but rather the interaction of race and ethnicity, as recommended by the *Ethnic racial identity in the 21*^*st*^ *Century Study Group* (Umana-Taylor et al., 2014). ERI can be broadly defined as an individual’s sense of self in relation to their membership of their ethnic group (Phinney, 1990; Yip et al., 2019). It may reflect both positive and negative domains and may include dimensions of affirmation, exploration, commitment, resolution, public and private regard, and centrality (Macedo, Santiago, et al., 2019; Yip et al., 2019). Attitudes towards one’s ethnic group, referred to in the Multigroup Ethnic Identity Measure as “affirmation” (Dandy et al., 2008, p. 325), have been theorised to be an important and separate aspect of ethnic identity (Brittian et al., 2015; Corenblum & Armstrong, 2012; Macedo, Santiago, et al., 2019). In the context of lateral violence, and questions about an individual’s Aboriginality, measures of affirmation may be particularly important due to their similarity to Aboriginal conceptualisations of identity (Boladeras, 2002; Clark et al., 2016).

Aboriginal identity may act as a protective factor for SEWB (Bamblett, 2006; Dudgeon et al., 2000, 2017; Kelly et al., 2009). Furthermore, some researchers have highlighted the potential for ERI to act as a buffer against the effects of stereotyping, prejudice and discrimination experienced by members of minority groups (Cokley, 2007; Neblett et al., 2010), and these findings have been supported in Aboriginal children (Macedo, Santiago, et al., 2019). One study that utilised a sample from the Longitudinal Study of Indigenous Children (LSIC, Department of Social Services [DSS], 2019) found that a strong sense of ERI affirmation may mitigate the risk of poor SEWB due to exposure to racism (Macedo, Smithers, et al., 2019). Further, evidence indicates that the strength of identity may function as a protective factor against racism (G. H. Bodkin-Andrews et al., 2013; Mellor, 2004). Evidence is, however, mixed (Macedo, Smithers, et al., 2019; Paradies & Cunningham, 2009). Overall, the present study sought to offer quantitative findings that support qualitative studies on the protective role of identity in the face of lateral violence (Clark et al., 2016; Young et al., 2017).

### Gaps in the literature

Whilst there is an emerging research base that is framed within Indigenous epistemologies and understandings in this area, many researchers have highlighted historical literature that lacked Indigenous-input (G. Bodkin-Andrews et al., 2017; Geia et al., 2012; Ryder et al., 2019). The LSIC data represents a unique opportunity for researchers to respond to gaps in research on important issues within Aboriginal communities. Recently, for instance, an edited collection from Indigenous researchers was published about the potential for the LSIC data to explore how Aboriginal children grow up strong in contemporary Australia (Walter et al., 2017). Furthermore, the LSIC offers the potential to extend current knowledge about lateral violence on SEWB beyond adult populations (Bennett, 2015; Boladeras, 2002; Mcalister & Wheeler, 2018) and to investigate whether these findings hold relevance for Aboriginal children. Further, if they do, it is important to investigate the potential to mitigate the impact of lateral violence. The LSIC offers some potential to test these relationships with quantitative models and Indigenous child and adolescent populations.

### The current study

Based on the literature reviewed above, the current study had two main aims. The first was to explore whether a relationship existed between lateral violence and SEWB among Indigenous children. The second was to determine whether a strong sense of identity, or ERI affirmation, would act as a protective factor in the relationship between lateral violence and SEWB. Therefore, consistent with SIT’s claim that individuals define themselves and others according to the social categories they belong to, and that this categorisation is important to their SEWB (Turner et al., 1987), it was hypothesised that children with more exposure to lateral violence would show more SEWB difficulties, as evidenced by scores on SDQ emotional and behavioural difficulties subscales. This relationship will be tested while controlling for potentially confounding variables. Secondly, consistent with prior research about high ethnic-identity affirmation as moderating the relationship between racism and SEWB (Macedo, Smithers, et al., 2019), it was hypothesised that ERI affirmation will moderate the relationship between lateral violence and SEWB. Further, we predict that the effects of lateral violence will be smaller among children with high levels of ERI affirmation, in all SDQ domains. This relationship will also be tested while controlling for potentially confounding variables.

## Methods

### Study design

The current study involved a secondary analysis of data gained within the LSIC, a national survey managed by the Australian Government’s Department of Social Services (DSS, 2019; Thurber et al., 2015). The LSIC’s purposive sampling included Aboriginal and Torres Strait Islander children from 11 sites across Australia and included individuals living in remote communities and metropolitan cities. It used a cross-sequential design that involved two cohorts of children whose data was collected in bi-annual waves. The present analysis used data from the LSIC K cohort, these data were collected in Wave 8 in 2015, and in Wave 10 in 2017 of the study.

### Data collection procedures

The LSIC was initiated and funded by the Australian Government Department of Social Services (DSS, 2019) and guided by a Steering Committee of Indigenous and non-Indigenous people. The study sought to inform policy to improve Aboriginal and Torres Strait Islander children’s SEWB, by recruiting participants from 11 sites using addresses provided by Centrelink and Medicare Australia. Further details of the study are provided in Data User Guides (Department of Social Services, 2019). Indigenous research administration officers obtained information through questionnaire-guided interviews of the study child, their main caregiver, secondary caregiver and teacher. Written consent was obtained from all informants, and authorisation for data collection with the study child was provided by children’s caregiver(s). Ethical approval for the original study was obtained from the Australian Government Department of Health and Ethics Committee, and the current study received ethics approval from the University of Technology Sydney Human Research Ethics Committee (ETH21-5890).

### Participants

Data used in this study were drawn from *n* = 360 children from the K-Cohort of the LSIC. ERI data was collected at Wave 8 of the study in 2015 when children were aged 10.5–12 years old. Lateral violence and SEWB data was collected at Wave 10 of the study in 2016 when children were aged between 12.5 and 14 years old. The majority of participants (*n* = 314, 87.2%) identified as Aboriginal, the remainder identified as Torres Strait Islander, and slightly over half (*n* = 196, 54.4%) came from an area with a low Level of Relative Isolation (LORI; S. Zubrick et al., 2005). The mean child age when information on lateral violence was collected was 13.0 years (range 11.4–14.7 years; *SD* = 5.9 years) and the sample was split equally by sex (*n* = 180. 50% female). All information used was generated through child self-report.

### Measures

#### Demographic measures

The LSIC asked participants several demographic questions in Wave 10 including their sex, level of relative isolation and Aboriginal identification (DSS, 2019). These data were used to provide demographic characteristics of the study’s participants that are presented in Table 1.

**Table 1.** Characteristics of study participants.

		n	%
**Categorical Variables**			
Sex	male	180	50
	female	180	50
LORI	None	114	31.7
	Low	196	54.4
	Moderate	25	6.9
	High/Extreme	25	6.9
Identification of Aboriginality/	Aboriginal	314	87.2
Torres Strait Islander	Torres Strait Islander	24	6.7
	Both Aboriginal and Torres Strait Islander	22	6.1
Ethnic-Racial Identity Dichotomised Score	High ERI	173	48.1
	Low ERI	187	51.9
**Continuous Variables**			
		mean	SD
Age at Wave 8 (months)		155.30	5.01
Exposure to lateral violence		5.25	1.57
**Ethnic-Racial Identity**			
ERI feels good		1.35	0,86
ERI wants to share		2.62	1.71
ERI feels safe		1.50	1.02
ERI wants people to know		1.88	1.34
**Strengths and Difficulties Questionnaire**			
SDQ Emotion		7.56	3.15
SDQ Conduct		7.65	2.75
SDQ Hyperactivity		9.88	3.08
SDQ Peer Problems		9.29	2.81
SDQ Prosocial		11.97	3.31
SDQ Total Difficulties		34.39	10.73

#### Exposure to lateral violence

There are no existing psychometric assessment measures of lateral violence (Webster & Clark, 2024). In the LSIC, experience of lateral violence was assessed by asking study children the following question: “In the past 12 months, how often did some Aboriginal or Torres Strait Islander people say you don’t look Aboriginal and/or Torres Strait Islander?” Participants were asked to rate their responses on a 6-point Likert scale (1 = Always; 2 = Most of the time; 3 = Fair Bit; 4 = Little Bit; 5 = Not Much; 6 = Never); scores were reverse-coded for ease of interpretation. This single item assessed study children’s exposure to lateral violence and is presented in Table 1.

#### Ethnic-racial identity affirmation

Study children answered a set of four questions regarding their ERI at school; these questions were as follows: 1) “I feel good about being Aboriginal and/or Torres Strait Islander in class”, 2) “I want to share (tell others) things about being Aboriginal and/or Torres Strait Islander in class”; 3) “I feel safe about being Aboriginal and/or Torres Strait Islander in class”; and 4) “I like people to know I am Aboriginal and/or Torres Strait Islander in class”. Participants were asked to rate their responses on a 6-point Likert scale (1 = Yes (Always), 2 = Yes (Most of the time), 3 = Sometimes (Fair bit), 4 = Sometimes (Little bit), 5 = No (Not much) and 6 = No (Never)). Consistent with Macedo, Smithers, et al. (2019), who found that the item reliability was satisfactory (α = 0.72), a single dichotomised variable was created for different levels of ERI affirmation. Children who answered (1) Yes (Always) and (2) Yes (Most of the time) to all four questions were included in the “High ERI affirmation” category.

#### Strengths and difficulties questionnaire (SDQ)

Child social and emotional outcomes were assessed by the SDQ (Goodman, 1997), a 25-item behavioural screening questionnaire for use with children aged 2–17 years. The questionnaire assessed child emotional and behavioural functioning across five subscales: emotional symptoms, conduct problems, hyperactivity, peer problems and prosocial behaviour. Each subscale was composed of five items, and scores on the emotional symptoms, conduct problems, hyperactivity and peer problems were summed to produce a total difficulties score (0–40). Participants were asked to respond to the measure in the LSIC on the basis that likert-type items were true (coded as 0), somewhat true (coded as 1), or certainly true (coded as 2). A score-range from 3 to 10 was obtained on each subscale with higher scores indicative of greater difficulties. Prior research in urban Aboriginal children (aged 4–17 years) samples indicated that the measure had good internal consistency for all subscales: SDQ total *α* = 0.85; emotional symptoms *α* = 0.70; conduct problems *α* = 0.78; hyperactivity *α* = 0.79; and prosocial *α* = 0.78 (Williamson et al., 2014). The peer problems subscale was, however, previously reported as poor; *α* = 0.47 (Williamson et al., 2014). Alphas obtained in the current study were calculated as: SDQ total *α* = 0.69; emotional symptoms *α* = 0.68; conduct problems *α* = 0.63; hyperactivity *α* = 0.64; peer problems *α* = 0.53; and prosocial *α* = 0.52.

#### Confounding variables

Demographic characteristics and socio-economic status were used to adjust for potential bias due to confounding. Confounding variables were conceptualised as a common cause of the exposure variables and the outcome variables (Greenland & Morgenstern, 2001). Within the literature among Aboriginal Australian children, multiple socio-economic and demographic characteristics are potentially confounding (Priest et al., 2013; Shepherd et al., 2012). Child sex, child age (years) and level of relative geographic isolation were included in the models. Child sex and age were provided in wave 10. The Level of Relative Isolation (LORI) is a measure of geographic remoteness (S. R. Zubrick et al., 2004) is based on the Accessibility/Remoteness Index of Australia and calculated based on relative distance to service centres. LORI categories range from “No isolation” (such as a metropolitan area), “Low isolation”, “Moderate isolation”, “High isolation” and “Extreme isolation”.

## Results

**Figure 1.**
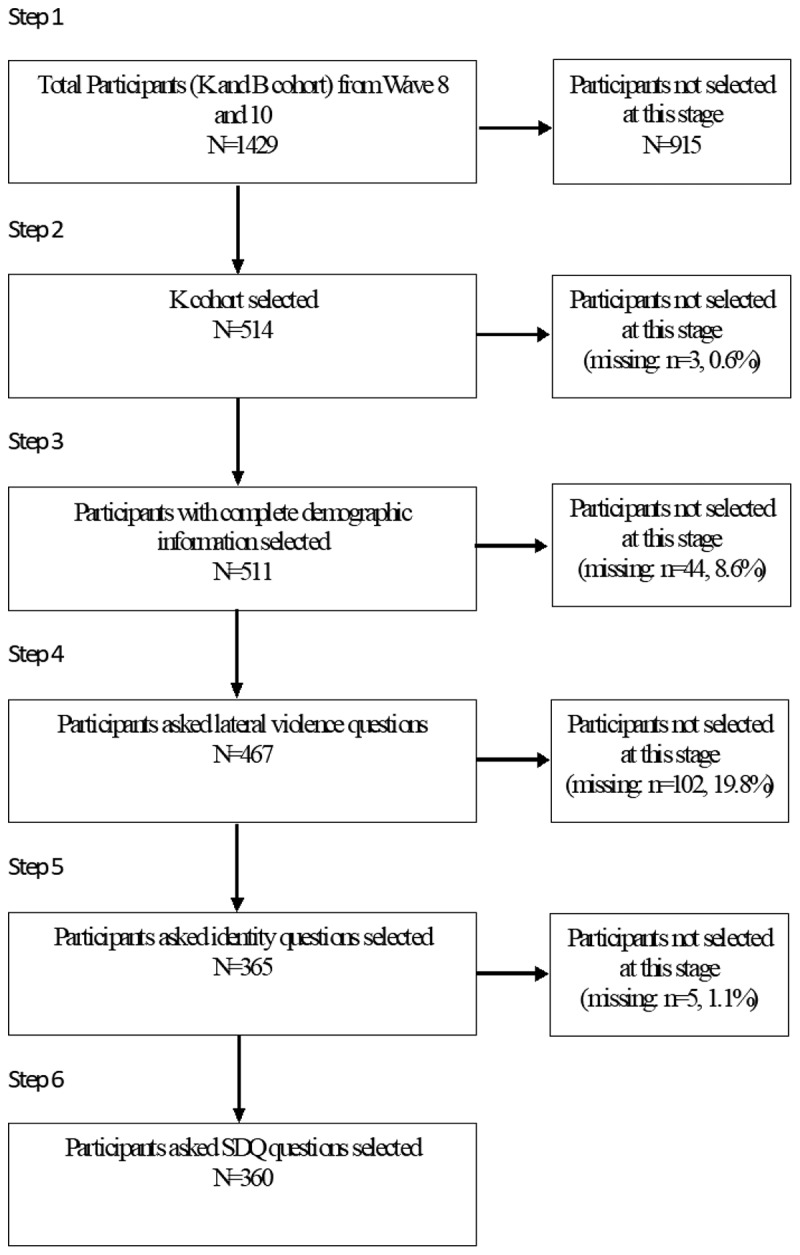
Identification and management of missing data.

**Figure 2.**
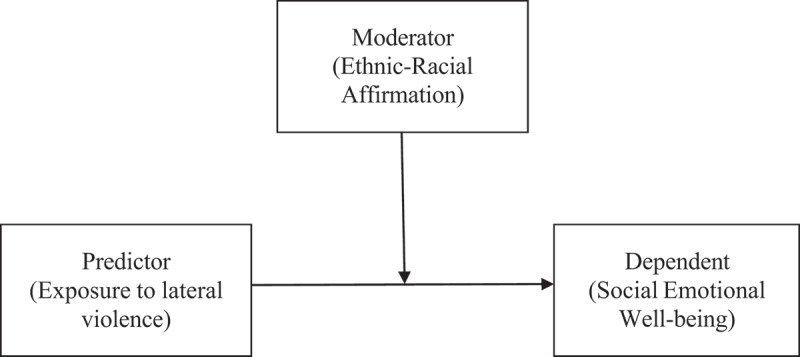
A conceptual model of moderation tested in hypothesis 2.

Prior to conducting analyses, results of the SDQ were converted from the range 0–2 to the range 1–3 and reverse items were reverse coded. The researchers undertook basic data screening to ensure assumptions of linearity, homoscedasticity, the independence of observations and normality of distribution were satisfied. The conduct subscale of the SDQ did not meet the assumptions of independence of observations (Durbin–Watson *p* < .001). Therefore, a logarithmic transformation was undertaken, and these results reported. All participants who had responses for all variables of interest were included in the analysis and a table accounting for the process of identifying and managing missing data is presented in Figure 1. Little’s MCAR test (Little, 1988) was not statistically significant, indicating that missing data was missing completely at random (χ^2^ (178) = 194.81, *p* = .184). The final analysis contained 360 participants. All data analyses were completed using the statistical package IBM SPSS Statistics version 23.0 (IBM Corp, 2015), or PROCESS Macro for SPSS, version 3.5.3 (Hayes, 2022). To test hypothesis 1, General Linear Models were used to determine if exposure to lateral violence had a main effect on scores on each SDQ subscale. To test hypothesis 2, moderation analyses were used to determine if ERI affirmation had a moderating influence on the relationship between exposure to lateral violence and scores on SDQ subscales (i.e., interaction between ERI affirmation and lateral violence exposure). A conceptual model of moderation is presented in Figure 2.

### Preliminary analyses

Approximately one-third of sample children (*n* = 105, 29%) reported some level of exposure to lateral violence. Consistent with cut points for the threshold for emotional and behavioural difficulties, between *n* = 52 and *n* = 120 (14.4% and 33.3% respectively) of sample children reported difficulties in any one of the SEWB domains. Slightly below half the children (*n* = 173, 48.1%) presented with high ERI affirmation. Table 1 presents demographic information of the sample and responses to experimental variables. Pearson’s correlations between continuous variables are presented in Table 2. A small but statistically significant negative correlation was evident between exposure to lateral violence and mean scores on all SDQ subscales, except the SDQ prosocial subscale which was negative and non-significant.

**Table 2.** Bivariate correlations between continuous variables.

	$\overset{ˉ}{x}$	SD	Age	ELV	ERI 1	ERI 2	ERI3	ERI4	SDQ Emot	SDQ Cond	SDQ Hyper	SDQ Peer	SDQ Pro	SDQ TD
Age (Mths)	155.89	5.91												
ELV	5.25	1.57	−.019											
ERI-1	1.35	0.86	.063	−.002										
ERI-2	2.62	1.71	−.040	.001	.256**									
ERI-3	1.50	1.02	.074	.014	.498**	.244**								
ERI-4	1.88	1.38	−.001	.035	.371**	.489**	.365**							
SDQ-E	7.56	3.15	.047	−.123**	.022	−.029	−.032	.013						
SDQ-C	7.65	2.58	.005	−.112*	.016	.038	.017	.032	.787**					
SDQ-H	9.88	2.88	.024	−.087*	−.010	.045	.003	.032	.712**	.795**				
SDQ-P	9.29	2.63	.064	−.097*	.034	.066	−.011	.055	.730**	.842**	.777**			
SDQ-Pro	11.97	3.31	.003	−.019	−.014	.001	−.044	−.083	.514**	.646**	.718**	.760**		
SDQ-TD	34.39	10.73	.039	−.115*	.017	.031	−.007	.036	.890**	.936**	.903**	.915**	.722**	

ELV = Experienced Lateral Violence; ERI = Ethnic-racial identity; SDQ = Strengths and Difficulties Questionnaire.

**Correlation is significant at the 0.01 level (1-tailed).

*Correlation is significant at the 0.05 level (1-tailed).

### Main effects for total SDQ difficulties scores

A significant effect of exposure to lateral violence on total SDQ difficulties was evident after controlling for the effects of age, sex and LORI, *F*(1,353) = 27.966, *p* < .05. Results indicated that a 1-unit increase in exposure to lateral violence was associated with a 1.7 unit increase in total SDQ difficulties scores (*b* = 1.687, 95% *CI* [1.059–2.314], *p* < .05).

### Main effects for SDQ subscales

#### Emotional difficulties

A significant effect of exposure to lateral violence on total emotional difficulties was evident after controlling for the effects of age, sex and LORI, *F*(1,353) = 12.521, *p* < .05. Results indicated that a 1-unit increase in exposure to lateral violence was associated with a 0.419 unit increase in emotional difficulties scores (*b* = 0.419, 95%*CI* [0.186–0.652], *p* < .05).

#### Conduct difficulties

A logarithmic transformation was undertaken on the conduct data and these data revealed a significant effect of exposure to lateral violence on total conduct difficulties after controlling for the effects of age, sex and LORI, *F*(1,273) = 6.625, *p* < .05. These results suggest that a 1-unit increase in exposure to lateral violence was associated with a 0.038 unit increase in conduct difficulties scores (*b* = 0.038, 95%*CI* [0.009, 0.066], *p* < .05). There was a significant effect of sex on conduct difficulties after controlling for the effects of lateral violence, age and LORI, *F*(1,273) = 8.716, *p* < .05. Compared to females, males were associated with a 0.095 unit increase in conduct difficulties scores (*b*= –0.095, 95%*CI* [0.032, 0.159], *p* < .05).

#### Hyperactive difficulties

A significant effect of exposure to lateral violence on total hyperactive difficulties was evident after controlling for the effects of age, sex and LORI, *F*(1,353) = 17.498, *p* < .05. A 1-unit increase in exposure to lateral violence was associated with a 0.49 unit increase in hyperactive difficulties scores (*b* = 0.488, 95% *CI* [0.259, 0.717], *p* < .05).

#### Peer difficulties

A significant effect of exposure to lateral violence on total peer difficulties after controlling for the effects of age, sex and LORI, *F*(1,353) = 15.816, *p* < .05. A 1-unit increase in exposure to lateral violence was associated with a 0.35 unit increase in peer difficulties scores (*b* = 0.353, 95% *CI* [0.178, 0.528], *p* < .05).

### Moderation analyses

**Figure 3.**
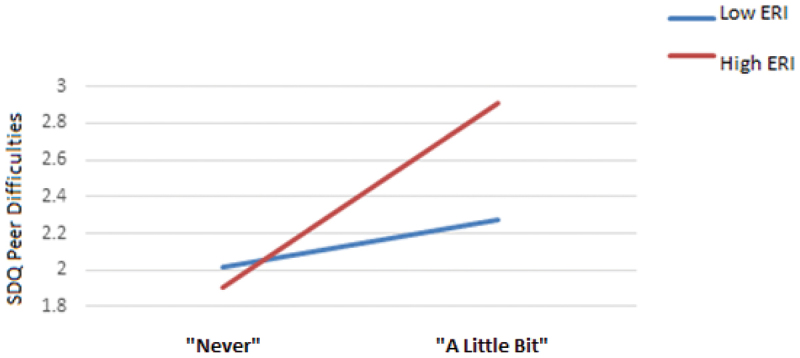
The moderating effect of ERI affirmation on the relationship between lateral violence and peer difficulties.

A simple moderation analysis, controlling for participants’ age, sex and LORI, was conducted for each SDQ subscale and the SDQ total difficulties score separately. Our expectation that ERI affirmation would moderate the relationship between lateral violence and SDQ subscales held for the peer difficulties subscale only, *b* =.371, 95% *CI* [0.019, 0.724], *t* = 2.074, *p* < .05, but not for the remaining SDQ subscales or SDQ total difficulties. Figure 3 offers a graphical account of this relationship which suggests that lateral violence and SDQ peer difficulties scores are moderated by ERI affirmation even when accounting for age, sex and LORI. When ERI affirmation is low, a non-significant, positive relationship between exposure to lateral violence and SDQ peer difficulties scores is evident, *b* = 0.131, 95% *CI* [−0.141, 0.402], *t* = 0.946, *p* =.345. By contrast, high ERI affirmation resulted in a positive and statistically significant relationship between exposure to lateral violence and SDQ peer difficulties scores, *b* = 0.502, 95% *CI* [0.277, 0.727], *t* = 4.389, *p* < .05.

## Discussion

This study sought to extend our understanding of the interactive effects of Indigenous identity and lateral violence on youth adjustment in Aboriginal and Torres Strait Islander children and the potential for ERI affirmation to act as a protective factor in this relationship. Our expectation that increased exposure to lateral violence would be associated with increased difficulties in all SDQ domains was supported with lateral violence demonstrated to have a significant negative effect on total SDQ difficulties, and all SDQ subscales except the prosocial subscale. The effects observed may be explained by SIT’s account of the negative impacts of lateral violence on an individuals’ overall SEWB as a result of other Aboriginal people questioning or denying their membership of their in-group (Corenblum & Armstrong, 2012). Further, the interplay of multiple social identities is likely to be more salient for those who are multi-racial, or those who lack physical characteristics associated with Aboriginality (Coffin, 2011). For instance, two adolescents with an Aboriginal parent – and a non-Indigenous parent – might experience lateral violence differently, depending on numerous factors, including the ethnicity of their other parent and their physical appearance.

Results partially supported our second hypothesis that ERI affirmation would moderate the relationship between exposure to lateral violence and SEWB, although this was in the opposite direction to that expected. We found a moderating effect of ERI affirmation on the relationship between exposure to lateral violence and peer difficulties such that increased exposure to lateral violence was associated with an increase in reported peer problems and this relationship was strengthened for the high ERI affirmation group. These results should be interpreted with caution, however, as the internal consistency of the SDQ Peer problems sub-scale has previously been reported as poor when used with Aboriginal people (Williamson et al., 2014).

Regardless, it may be argued that the results of this study were consistent with Macedo, Smithers, et al. (2019) who found a similar moderating effect of ERI affirmation on the relationship between exposure to racism and peer problems. In that research, risk for peer problems in children in the high ERI affirmation group doubled, although the moderating effect was in the opposite direction for emotional, conduct and hyperactivity problems. Consistent with SIT, children in the high ERI affirmation group may consider their ERI more salient than those in the low ERI affirmation group (Douglass et al., 2016). In turn, this higher level of ERI affirmation, and its expression, may put the Indigenous children at greater risk of experiencing questions about their Indigeneity from Indigenous peers. This may pose a greater threat to their identity, associated SEWB and interpersonal interactions (Coffin, 2011). Furthermore, SIT predicts that experiences of discrimination in relationship to one’s ERI group may function to increase the salience of one’s ERI affirmation, and this relationship may be more important for adolescents in the process of establishing their identity (T. B. Smith & Silva, 2011).

### Strengths and limitations of the current study

The current study was the first we are aware of to utilise quantitative methods to understand the negative effects of lateral violence on Indigenous children’s SEWB. Our results are consistent with a wider literature on the negative impact of exposure to lateral violence on SEWB, particularly for Aboriginal children. These findings are further backed by the observation that the LSIC study has a large sample size, and although the LSIC cannot be considered representative of the Aboriginal child population in Australia (due to its non-random purposive sampling design), the range of geographical locations included in the LSIC study provides more coverage of Aboriginal and Torres Strait Islander children than previous studies.

Perhaps, the most important finding of this study was that an increase in exposure to lateral violence was associated with increased difficulties in all SDQ domains. This may be attributable to the understanding from that lateral violence negatively impacts an individual’s overall SEWB because of others questioning or denying their membership of the (Aboriginal) in-group (Corenblum & Armstrong, 2012). We do not conclude that ERI is a factor of risk for poor outcomes. Rather, it appears that high ERI affirmation may function to challenge established racial norms in Australia, in this context in schools, and that there may be a reciprocal relationship between the voracity with which one holds their ERI affirmation and their experience. The moderation effect we found suggests that children high in ERI affirmation have greater vulnerability to the negative impacts of lateral violence than those with lower affirmation. There may, of course, be significant additional factors at play in school settings, and it is appropriate that future research further explore additional dimensions of ERI and additional contextual factors (Macedo, Santiago, et al., 2019; Yip et al., 2019).

Some limitations associated with the present study must be acknowledged. Firstly, the SDQ may not provide a comprehensive understanding of SEWB, particularly for those Indigenous children who live in more remote areas, as subscale reliabilities have been found to decline as the level of remoteness increases (S. R. Zubrick et al., 2006). The SDQ’s acceptability was established within a population of Aboriginal children living in urban areas, which is not fully representative of the Aboriginal child population (Williamson et al., 2014). Further, the SDQ is a Western measure of SEWB, and may not fully capture Indigenous SEWB – future studies may, for instance, operationalise SEWB as defined by the Bardi scholar Dudgeon et al. (2017) model.

Secondly, although the ERI affirmation measure has been validated among a sample of Australian Aboriginal children aged 10–12 years (Macedo, Santiago, et al., 2019), this study utilised the LSIC data, indicating that more research needs to be conducted on the acceptability of this measure across a more representative sample. Additionally, the ERI affirmation measure in the LSIC data is limited to children’s experiences in school, which may limit the generalisability of these results to other contexts. Considering the ERI affirmation measure was comprised of items that were constructed with a high level of community engagement; however, it may be considered a culturally appropriate measure (Thurber et al., 2015). Considering the current study utilised a measure that encapsulated one domain of Phinney’s (1990) ERI, it would be beneficial for future studies to explore the different domains of ERI to identify potential variations in the relationship between Aboriginal children’s ERI and well-being (Schwartz et al., 2014). For example, within the seminal work of Wadjuk Noongar scholar C. S. Kickett-Tucker (2009), it was found through a thematic analysis of interviews with Aboriginal children, that the children held approximately 30 diverse themes that incorporate their sense of identity and self. Additional dimensions of ERI have also been identified and warrant exploration with Indigenous Australian children (Yip et al., 2019)

In the present study, lateral violence was assessed using a single LSIC item in a sample of 12.5–14-year-olds . This may limit the studies ability to assess the presence and nature of lateral violence accurately, and it may limit the generalisability of the study findings, especially later in adolescence when lateral violence and identity formation may be more pronounced. It is also noteworthy that the majority (70.8%) of study children reported having *never* been exposed to lateral violence based on their physical characteristics. Future studies could further explore the nature of children’s exposure to lateral violence, including variables that may explain whether a child is questioned on their Aboriginality. For example, research that explores lateral violence in terms of skin colour and phenotypical features, as well as other personal characteristics, will assist in establishing the prevalence and implications of lateral violence for this population. The findings of the present study do, however, provide useful insights for public health, and psychological treatments for Aboriginal children. The negative impact of lateral violence on Aboriginal children’s SEWB, as well as the potential for a strong sense of ERI affirmation to act as a moderator in this relationship, particularly within a school setting, can guide preventative and early intervention approaches to improving the mental health of Indigenous children.

## Conclusion

This study extended our understanding of the relationship between Indigenous identity and lateral violence and the implications these influences hold for Aboriginal and Torres Strait Islander children. Despite many qualitative studies on the impact of lateral violence on Aboriginal children’s SEWB, quantitative studies in this area are rare. Our study contributed an understanding of the impact of lateral violence on the SEWB of Aboriginal and Torres Strait Islander children, and the potential for ERI affirmation to act as a protective factor in this relationship. Importantly, higher ERI affirmation in combination with exposure to lateral violence was shown to be associated with more emotional and behavioural difficulties in youth. We acknowledge that children who are highly engaged with their cultural identity, and who may be questioned or challenged about it, are likely to respond more vigorously than children who are less attached to their identity. The findings of this study are discussed in relationship to their potential to assist in public policy and clinical practice.

## Data Availability

The data that support the findings of this study pertain to the Longitudinal Study of Australian Children and are available from Australian Government’s Department of Social Services (DSS). Restrictions apply to the availability of these data, which were used under licence for this study.

## Public significance statement

Lateral violence is the potential for members of a marginalised group to engage in practices that are harmful to other members of that group. This study extended understanding of lateral violence in relationship to Indigenous identity by showing that these factors may be associated with poor outcomes for Aboriginal and Torres Strait Islander youth. Further, findings suggested that children who have high Ethnic Racial Identity, and who may be challenged about their identity, are likely to respond more vigorously than children who are less attached to that identity.

## Position statement

The researchers of this study recognise that Aboriginal and Torres Strait Islander communities each have their own separate cultures, beliefs, histories and values. Thus, to reflect the complexity and importance of not combining all Indigenous people, the term Aboriginal will be used when referring generally to the Aboriginal population, and the term Aboriginal and Torres Strait Islander or Indigenous Australians will be used when referring specifically to research involving both Aboriginal and Torres Strait Islander people. In addition, when writing within Indigenous contexts, it is becoming increasingly important to note the cultural positioning of the researchers themselves, and to also identify Indigenous-led research (Phillips et al., 2007). Three of the authors within this paper identify as Aboriginal and bring cultural perspectives and lived experiences to the research topic (Mcalister–Wiradjuri, Brockman–Worimi and Bodkin–Andrews–D’harawal). Two of the authors, whilst non-Indigenous (Rogers and McAloon), bring considerable clinical and statistical experience to this research.
